# The effect of different types of insoles or shoe modifications on medial loading of the knee in persons with medial knee osteoarthritis: a randomised trial

**DOI:** 10.1002/jor.22947

**Published:** 2015-06-03

**Authors:** Richard K. Jones, Graham J. Chapman, Matthew J. Parkes, Laura. Forsythe, David T. Felson

**Affiliations:** ^1^ School of Health SciencesUniversity of SalfordSalfordUnited Kingdom; ^2^ Clinical Biomechanics & Physical Medicine SectionLeeds Institute of Rheumatic and Musculoskeletal Medicine (LIRMM) and Leeds NIHR Biomedical Research UnitUniversity of LeedsLeedsUnited Kingdom; ^3^ Arthritis Research UK Centre of Excellence in EpidemiologyCentre for Musculoskeletal ResearchUniversity of ManchesterManchesterUnited Kingdom; ^4^ NIHR Manchester Musculoskeletal Biomedical Research UnitManchester Academic Health Sciences CentreManchesterUnited Kingdom; ^5^ Clinical Epidemiology UnitBoston University School of MedicineBostonMassachusetts

**Keywords:** knee osteoarthritis, footwear, lateral wedge insoles, adduction moment, walking

## Abstract

Many conservative treatments exist for medial knee osteoarthritis (OA) which aims to reduce the external knee adduction moment (EKAM). The objective of this study was to determine the difference between different shoes and lateral wedge insoles on EKAM, knee adduction angular impulse (KAAI), external knee flexion moment, pain, and comfort when walking in individuals with medial knee OA. Seventy individuals with medial knee OA underwent three‐dimensional walking gait analysis in five conditions (barefoot, control shoe, typical wedge, supported wedge, and mobility shoe) with pain and comfort recorded concurrently. The change in EKAM, KAAI, external knee flexion moment, pain, and comfort were assessed using multiple linear regressions and pairwise comparisons. Compared with the control shoe, lateral wedge insoles and barefoot walking significantly reduced early stance EKAM and KAAI. The mobility shoe showed no effect. A significant reduction in latter stance EKAM was seen in the lateral wedge insoles compared to the other conditions, with only the barefoot condition reducing the external knee flexion moment. However, the mobility shoe showed significant immediate knee pain reduction and improved comfort scores. Different lateral wedge insoles show comparable reductions in medial knee loading and in our study, the mobility shoe did not affect medial loading. © 2015 The Authors. *Journal of Orthopaedic Research* Published by Wiley Periodicals, Inc. J Orthop Res 33:1646–1654, 2015.

Knee osteoarthritis (OA) is the most common form of OA and is the leading cause of pain and disability in older adults.^1^ At the current time, there is no cure for knee OA and therefore non‐surgical conservative management is at the forefront of the treatment for the disease. In the UK, National Institute of Clinical Excellence (NICE) guidelines recommend conservative management techniques such as footwear and insoles to be part of the management of the condition.^2^ The medial compartment of the knee joint is more often affected than the lateral compartment.^3^

Dynamic joint loading has been implicated both in the development of knee pain associated with OA^4^ and the progression of the disease.^5^ During walking the ground reaction force passes medial to the knee in the frontal plane, this creates a moment that adducts the tibia relative to the femur, with the peak load on the medial compartment almost 2.5 times more than that on the lateral compartment.^6^ The external knee adduction moment (EKAM), captured from three‐dimensional motion analysis and inverse dynamics, is a valid and reliable proxy representing dynamic load distribution and is the primary mechanism, along with the external knee flexion moment, for the majority of compressive load in the joint.^6^, ^7^, ^8^ The EKAM typically has an early stance peak (first) and a late stance peak (second) with the first peak always higher than healthy controls regardless of severity, whereas the second peak is only higher in the more severe individuals.^9^ Therefore, given the target population for conservative management (mild and moderate knee OA), the primary parameters of interest are the first peak in the EKAM and also the knee adduction angular impulse (KAAI), which is the area under the adduction curve.^10^ These two parameters have been demonstrated to be related to severity^11^ to structural features of OA and to progression.^12^, ^13^ Therefore, reducing the EKAM during walking and other activities could be effective in delaying progression in medial knee OA.

Many unloading strategies are available including proximal and distal gait adaptations, direct orthotic management at the knee such as valgus knee braces,^14^, ^15^ or indirectly at the foot and ankle interface such as shoes/footwear and foot orthoses such as lateral wedge insoles.^16^, ^17^, ^18^ The latter are popular as they are typically inexpensive with good adherence to treatment. Different types of shoes and orthotics have been shown to reduce the EKAM in knee OA trials^16^, ^18^, ^19^ but these have not been directly compared in terms of their effects on medial knee loading and clinical responses. Further, in recent studies directly measured medial compressive loads have been shown to be affected by the magnitude of the external knee flexion moment^20^ in that a reduction in EKAM may not correspond with a true reduction in medial loads if a corresponding increase in knee flexion moment was seen. The literature on the different effects of lateral wedge insoles and shoe modifications on the knee flexion moment has also not been fully described.

There is also not one type of lateral wedge insole, but rather several types such as heel only, full length, and full length insoles with medial support, also with different angulations of lateral incline. In this study we chose to investigate a full length lateral wedge insole (typical) as these have been found to be better than heel only wedges^21^ and also one with a medial arch support (supported), as this was previously found to be better functionally for the foot and ankle and more comfortable.^22^ We have demonstrated in a previous paper the effects on early stance peak EKAM and external knee flexion moment of these two different types of lateral wedge insole.^18^ In addition, other footwear based approaches to lowering medial load have been proposed. One such shoe treatment which aims to mimic barefoot walking during gait,^16^ which is perceived as the best walking style for reducing medial loading, has been developed and recommended as efficacious for medial knee OA. These shoes have not been directly compared with traditional lateral wedge insoles in terms of their effects on medial knee load.

Understanding which treatment reduces medial loading and reduces pain may provide guidance in terms of which, if any, of these treatments is most likely to be efficacious for medial knee OA. The objectives of this study were to determine which of several different conservative treatments (barefoot, shoes, and insoles) most lowers the EKAM during walking, to determine if any concurrent changes occur in the external knee flexion moment and to compare the degree of immediate knee pain reduction and comfort during usage.

## METHODS

The study is a Level 1 evidence randomised clinical trial (ISCRTN 83706683) whereby ethical approval was obtained from the North West Research Ethics Committee (09/H1013/51).

### Participants

Participants with knee pain were recruited from the following sources: orthopaedic/physiotherapy clinics and advertisements in local media. The eligibility criteria for participation in the study were aged 45 years and above, medial tibiofemoral OA with radiographs demonstrating Kellgren and Lawrence grade 2 or 3 in the affected painful knee with medial greater than lateral joint space narrowing, and at least mild pain during walking on a flat surface during the last week assessed by the KOOS pain subscale (P5).^23^ Radiographs were generally acquired as part of the patient's routine care and were read by an experienced academically based musculoskeletal radiologist according to the OARSI atlas.^24^ When no radiographs were available, we accepted evidence from recent arthroscopies or knee MRI's as providing sufficient information to evaluate eligibility. Patients were excluded if they presented with pain more localised to the patellofemoral joint on examination than medial joint (wedge inserts are not appropriate for disease in this compartment and lowering the EKAM may make them worse), had tricompartmental knee OA or grade 1 or grade 4 tibiofemoral OA on the Kellgren and Lawrence scale. Other exclusions included a history of high tibial osteotomy or other realignment surgery, total knee replacement on the affected side, or any foot and ankle problems**,** such as painful hallux valgus; plantar fasciitis; peripheral neuropathy or any foot and ankle pain, that contraindicated the use of the load modifying footwear interventions. In addition, participants were excluded if they had severe coexisting medical morbidities or used orthoses prescribed by a podiatrist or orthotist. Eligible participants were invited to attend the gait laboratory where informed consent was obtained.

### Interventions

**Figure 1:**
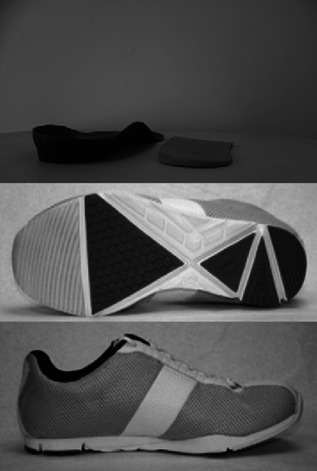
Lateral wedge insoles and mobility shoes.

The analyses were conducted in the context of a single visit randomised trial. We tested five conditions: barefoot, a flat soled shoe (Ecco Zen) (control), two different lateral wedge insoles each which have been shown to reduce EKAM in patients with medial knee OA^18^, ^25^ and a mobility shoe^16^ meant to mimic barefoot walking. Both lateral wedge insoles consisted of a 5 degree lateral wedge. The major difference between the lateral wedge insoles was that one had medial support (referred to hereafter as the “supported” wedge^18^ whereas the other had no medial support (the “typical” wedge).^25^ During the trial, these lateral wedges were inserted into the flat‐soled control shoe with participants having a minimum of 5 min familiarisation period to the condition. The mobility shoe was a flexible grooved shoe^16^ (see Fig. 1).

### Protocol

All participants underwent gait analysis in all of the conditions. The order of presentation of the different conditions was randomised prior to participants' enrolment using computer‐generated permutations (using http://www.randomization.com/). Patients walked at their self‐selected speed in all conditions. Upon completing each treatment, participants were asked to compare the knee pain experienced under that treatment while walking to pain when wearing their own shoes by scoring this pain on a 5‐point Likert scale ranging from −2 (indicating much better pain compared to the participants' normal shoes) to +2, (indicating much worse pain compared to the participant's normal shoes). Additionally, we asked participants to rate each condition's comfort, in comparison to their normal everyday shoes. This was measured on a 10 cm VAS, with scores ranging from −5 (much less comfortable than the participants' normal shoes), to +5 (much more comfortable than the participant's normal shoes). All outcomes were measured in all five study conditions (control shoe, typical lateral wedge, supported lateral wedge, mobility shoe, and barefoot.)

A 16 camera Qualisys OQUS3 motion analysis system operating at 100 Hz and four AMTI force plates operating at 200 Hz were used to measure kinematics and kinetics during the trials. Each participant completed a minimum of three successful trials at a self‐selected walking speed. The CAST marker set technique^26^ was employed whereby rigid clusters of four non‐orthogonal markers were positioned over the lateral shank, lateral thigh, and sacrum to track the movements of the limbs. Retroreflective markers were glued securely to the control shoes with the foot modelled as a rigid segment. A reference trial was conducted where retroreflective markers were placed on bony landmarks specifying their location in relation to the clusters and to approximate joint centre. Ankle and knee joint centres were calculated as midpoints between the malleoli and femoral epicondyles respectively. The hip joint centre was calculated using the regression model of Bell et al.^27^ based on the anterior and posterior superior iliac spine markers. Using an inverse dynamic approach Visual 3D (C‐Motion, Rockville, Maryland) we calculated the EKAM and sagittal plane external knee flexion moment (KFM) during stance phase for all of the trials and conditions. A custom Matlab (Matlab, USA) programme was used to extract the peak EKAM and KFM during early stance (up to 50% of stance phase) and the peak latter stance EKAM (from 50% of stance phase) and to calculate the knee adduction angular impulse (KAAI),^9^ which is the area under the adduction moment curve during the entire stance phase of gait. EKAMs and KFMs were normalised to participant's mass (Nm/kg) with the KAAI normalised to participant's mass and stance time (Nm/kg s).

### **Data** Analysis

Multiple linear regression was used to test for differences in continuous outcomes of interest, between the 5 different experimental conditions. We created four models, one for each of the gait outcomes of interest (EKAM [first and second peak] KAAI, and KFM). In each model, the predictor variable was the orthotic intervention, coded as “dummy variables”—giving 5 predictor variables in total, one for each condition). The control shoe condition was used as the reference group. The model also accounted for the repeated‐measures design of the study by including the participant ID as a panel variable. We used a Hausman specification test to check for the validity of using a random‐effects model, in preference to a fixed‐effects model of the same specification. The test did not show statistical significance and consequently, a random‐effects model was used. We checked for model fit by investigating residuals against fitted plots. Since model residuals appeared heteroskedastic, robust standard errors (using sandwich estimators) were used to improve estimates of standard errors. Post‐hoc pairwise contrasts were produced, using linear combinations of the beta coefficients from the model to test for differences in all possible comparisons of the orthotics conditions, with ten pairwise tests for each of the three outcomes considered (EKAM, KAAI, and maximum external flexion moment). To counter issues of multiple testing, confidence intervals and *p*‐values from these pairwise tests were adjusted using the Benjamini‐Hochberg procedure,^28^, ^29^ using a false discovery rate (FDR) of 0.05 (see supplementary material).

Because patient perceived change in knee pain was not normally distributed, we used Wilcoxon Sign Rank tests to investigate whether the distribution of patient‐perceived pain change ranks were equal to zero, in each orthotic condition separately.

Finally, for each condition, we measured if the patient‐perceived change in comfort was different from zero. To test this, we used a random‐effects linear regression model, with the participants' comfort ratings as the outcome variable. The predictor variable again was the orthotic intervention condition, coded as “dummy variables”, as in the EKAM/KAAI regression. We then combined the model intercept with the beta coefficients of each condition in turn. This tests if the mean comfort rating in each is equal to zero. Additionally, as both walking speed and knee flexion moment were considered potential confounding variables, we repeated the above models, with walking speed and external knee flexion moment added as additional covariates.

All statistical analysis was performed using the statistical software package Stata (version 13.1; Stata Corporation, College Station, TX), with an alpha level of 0.05 (two‐sided) for the assessment of statistical significance.

## RESULTS

**Figure 2:**
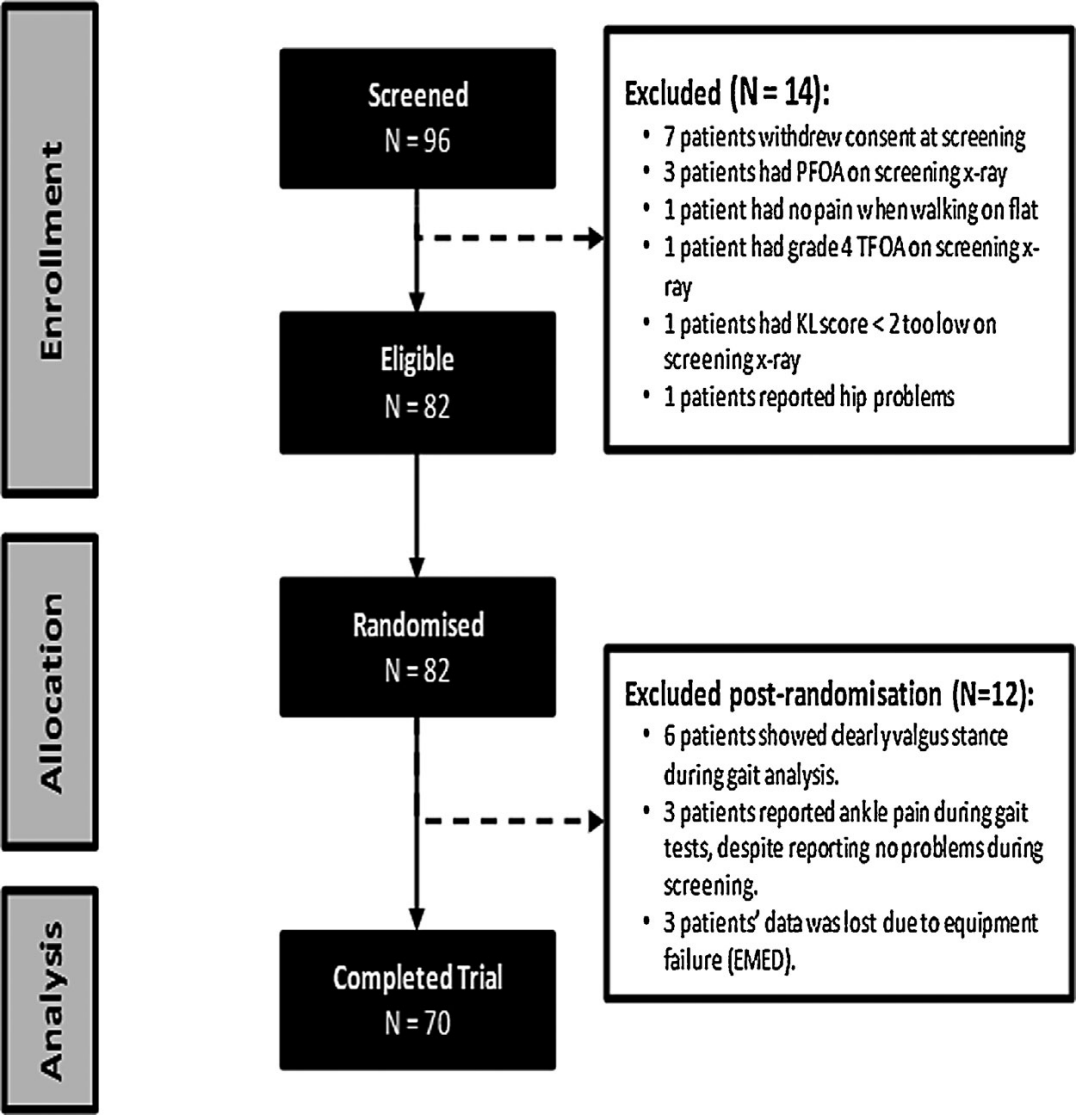
Consort Figure: those eligible/enrolled/randomised/studied.

The flow of participants into the study is shown in Figure 2. The characteristics of the 70 participants were: a mean age of 60.3 years (SD 9.6), mean BMI of 30.5 kg/m^2^ (SD 4.9), and 27 (38.6%) were female. Data on Kellgren‐Lawrence (K‐L) grades were available for 62 of the 70 study participants, and of these, the mean K‐L grade was 2.6 (SD 0.5). We reviewed recent knee arthroscopy reports or MRIs for 8 participants who did not have x‐rays prior to the study to ensure that these subjects also had medial > lateral cartilage loss and other features of OA.

**Figure 3:**
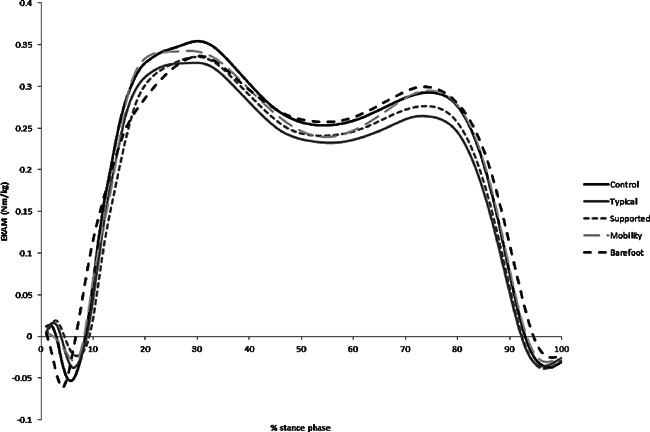
EKAM time series plots for the different conditions (*n* = 70).

When we examined the effects of the conditions on measures of medial loading (Tables 1 and 2, Fig. 3), we found that barefoot walking had the greatest effect on early stance peak EKAM, lowering it by −7.6% (*p *< 0.001 vs. control shoe). Both lateral wedges reduced the early stance peak EKAM by −5.9 and −5.6% (*p* = 0.001 vs. control shoe) for typical and supported respectively as we have previously reported.^18^ However, the mobility shoe did not produce a significant reduction in the early stance peak EKAM compared with the control shoe (−1.6%, *p* = 0.38).

**Table 1:** EKAM, KAAI, Knee Flexor moment, Comfort Rating (VAS), and Walking Speed by Condition

Condition	EKAM 1st Peak, Nm/kg Mean (SD)	EKAM 2nd Peak, Nm/kg Mean (SD)	KAAI, Nm/kg*s Mean (SD)	Knee Flexor Moment (KFM), Nm/kg Mean (SD)	Comfort Rating (‐5 to +5), Mean (SD)	Walking Speed, m/s Mean (SD)
Control shoe	0.39 (0.16)	0.33 (0.14)	0.16 (0.07)	0.61 (0.24)	‐0.24 (2.29)	1.08 (0.33)
Typical lateral wedge	0.37 (0.15)	0.30 (0.13)	0.14 (0.07)	0.61 (0.23)	0.84 (2.42)	1.08 (0.33)
Supported lateral wedge	0.37 (0.15)	0.31 (0.14)	0.15 (0.07)	0.62 (0.23)	1.35 (2.13)	1.09 (0.33)
Mobility shoe	0.39 (0.16)	0.32 (0.14)	0.15 (0.07)	0.60 (0.24)	2.40 (2.22)	1.11 (0.34)
Barefoot	0.36 (0.15)	0.33 (0.14)	0.15 (0.07)	0.57 (0.22)	0.48 (2.35)	1.04 (0.33)

**Table 2:** Effects of Study Footwear on Moments and Walking speed compared with control shoe

	EKAM 1st Peak		EKAM 2nd Peak		KAAI		Knee Flexor Moment (KFM)		Walking Speed (m/s)	
Condition	mean change (95% CI), p	% change	mean change (95% CI), p	% change	mean change (95% CI), p	% change	mean change (95% CI), p	% change	mean change (95% CI), p	% change
Typical lateral wedge	‐0.023 (‐0.035 to ‐0.012), <0.001***	‐5.85	‐0.028 (‐0.036 to ‐0.02), <0.001***	‐8.49	‐0.012 (‐0.016 to ‐0.009), <0.001***	‐7.95	‐0.002 (‐0.022 to 0.018), 0.818	‐0.39	0.003 (‐0.007 to 0.013), 0.558	0.28
Supported lateral wedge	‐0.022 (‐0.035 to ‐0.009), 0.001**	‐5.63	‐0.018 (‐0.026 to ‐0.01), <0.001***	‐5.52	‐0.009 (‐0.013 to ‐0.005), <0.001***	‐5.52	0.013 (‐0.004 to 0.03), 0.133	2.17	0.009 (‐0.002 to 0.019), 0.105	0.79
Mobility shoe	‐0.006 (‐0.021 to 0.008), 0.384	‐1.61	‐0.005 (‐0.015 to 0.005), 0.294	‐1.59	‐0.004 (‐0.009 to 0.001), 0.090	‐2.74	‐0.006 (‐0.029 to 0.017), 0.611	‐0.99	0.029 (0.018 to 0.039), <0.001***	2.65
Barefoot	‐0.03 (‐0.044 to ‐0.016), <0.001***	‐7.62	0.001 (‐0.011 to 0.013), 0.856	0.34	‐0.007 (‐0.013 to ‐0.001), 0.023*	‐4.30	‐0.035 (‐0.057 to ‐0.013), 0.002**	‐5.74	‐0.042 (‐0.056 to ‐0.028), <0.001***	‐3.89

Asterisks denote magnitude of p‐value as follows:*p <0.05; **p<0.01; ***p<0.001.

For the second peak in EKAM during late stance, both of the lateral wedge insoles significantly reduced the magnitude of this peak in comparison to the control condition. There was no difference in the mobility or barefoot conditions in comparison to the control condition. For the knee adduction angular impulse (KAAI), the barefoot condition and the two lateral wedge conditions were significantly different in comparison to the control condition (barefoot −4.3%, *p *= 0.023; typical wedge −7.95%, *p *< 0.001; supported wedge −5.5%, *p *< 0.001).

Pairwise comparisons (see supplementary material eTables S1–3) showed that there were no significant differences in the effects on the early stance peak in EKAM between each of the two lateral wedge conditions and barefoot walking. However, the early stance peak in EKAM in the barefoot condition was reduced significantly more than the mobility shoe (mean difference −0.024 Nm/kg, *p *= 0.004).

For the second peak in EKAM, both of the lateral wedge insoles had significantly greater reductions than the barefoot (typical wedge mean difference −0.029 Nm/kg, *p *< 0.01; supported wedge mean difference −0.019 Nm/kg, *p *= 0.004) and mobility (typical wedge mean difference −0.023 Nm/kg, *p *< 0.01; supported wedge mean difference −0.013 Nm/kg, *p *= 0.024) conditions. A larger second peak reduction in the typical wedge resulted in a significant reduction in KAAI in comparison to the mobility shoe (mean difference 0.008 Nm/kg s, *p *= 0.011).

In comparison with the control shoe and all other conditions, the barefoot condition had significant reductions in the maximum external knee flexion moment (KFM) (etable 3) during early stance. No other changes in external knee flexion moment were seen.

Compared with the control shoe, walking speed increased by 0.03m/s with the mobility shoe (95%CI 0.02–0.04, *p* < 0.001) and slowed by 0.04 m/s with barefoot walking (95%CI −0.05 to −0.03, *p *< 0.001), but with adjustment for walking speed, this did not affect the overall findings or their significance. Additional adjustment for external knee flexion moment changes also did not affect the differences seen between conditions in medial load measures.

**Figure 4:**
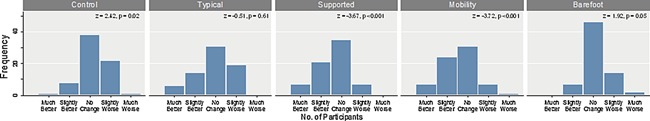
Participant rating of knee pain during use of each condition compared with knee pain using their own shoe.

In contrast with the findings with regard to medial loading, immediate reductions in knee pain were seen in two conditions: the supported (but not the typical) wedge (as reported previously)^17^ and the mobility shoe (both *p* < 0.001) (see Fig. 4). A significant worsening of knee pain was reported by patients for the control shoe (not the subject's own shoe) (*p* = 0.015) and barefoot walking (*p* = 0.054).

In terms of comfort, the control shoe was rated as less comfortable than the participant's everyday shoes (see Table 3). Even though the wedges were placed inside these control shoes, both lateral wedges were rated as more comfortable than everyday shoes as were the mobility shoes.

**Table 3:** Participants report of shoe/condition comfort

	Mean Comfort Rating compared with participants’ own shoe (10cm VAS, ‐5 to +5)
Condition	mean (95% CI), p
Control	‐0.243 (‐0.777 to 0.291), 0.373
Typical lateral wedge	0.844 (0.31 to 1.379), 0.002**
Supported lateral wedge	1.349 (0.814 to 1.883), <0.001***
Mobility shoe	2.403 (1.869 to 2.937), <0.001***
Barefoot	0.464 (‐0.074 to 1.002), 0.091

Asterisks denote magnitude of p‐value as follows:*p <0.05; **p<0.01; ***p<0.001.

*Negative value represents report that condition is less comfortable than current shoe whereas positive value represents report that condition is more comfortable. Wedges were placed inside control shoe*.

## DISCUSSION

To examine the effects of shoes and orthotics suggested as effective in unloading the medial knee compartment, we carried out a randomised trial, comparing these treatments. We found that barefoot walking and lateral wedge insoles all significantly reduced medial loading in the first part of stance phase with both of the lateral wedge insoles reducing medial loading during latter periods of stance. The two types of lateral wedge insoles showed roughly comparable effects on the knee adduction moment and impulse with only the barefoot walking significantly altering the sagittal moment. Although the mobility shoe did not reduce medial knee loading, participants reported that it diminished knee pain more than the typical wedge, control shoe, and barefoot, and was rated as more comfortable than the other treatments.

While lateral wedge inserts have not been shown to decrease knee pain in knee osteoarthritis,^30^ they do reduce medial loading. Since excess loading in the medial compartment contributes to knee pain and disease progression and since knee OA treatments that alter this are likely to be popular and inexpensive, further exploration of their possible effects is desirable. In that vein, our work suggests two important findings. First, they suggest that lateral wedge insoles reduce medial knee loading more than a control shoe throughout the whole of stance phase and significantly better than both barefoot walking and the mobility shoe during latter stance where the supported insole reduces immediate knee pain better than the typical device with increased comfort. Secondly, whist the mobility shoes did not reduce medial loading significantly, they were rated highly by participants for reductions in knee pain and comfort.

Barefoot walking was found to have the greatest reduction in EKAM in comparison to the control shoe and is in agreement with a previous study by Shakoor and Block.^31^ However, they found a greater reduction (−11.9%) in the peak EKAM (KAAI was not assessed in that study). Differences between our study and that of Shakoor and Block could have accounted for the difference in the magnitude of effect. We focused on the early stance first peak EKAM and not the peak EKAM (which is sometimes different) and we used one control shoe whereas they compared barefoot to a person's individual shoes. We found importantly that barefoot walking reduced medial loading during latter stance in comparison to the control shoe, but had increased medial loading in the latter period of stance in comparison to the lateral wedge insoles. This reduced latter stance reduction in EKAM in the lateral wedge insoles, whilst not directly related to severity or progression, would contribute to a greater reduction in the overall loading during stance phase (KAAI) which has been related to cartilage loss.^13^ Different shoes may differ in their effect on medial knee loading and our control shoe may have been more effective than some personal shoes in reducing knee medial loads. An exploration of types of personal shoes and their effects on knee loading was beyond the scope of this trial but this is an important next step to determine what role different footwear has on medial knee loading.

In agreement with Jones et al.,^22^ there was no change in the reduction of EKAM or KAAI with the two different lateral wedge insoles. This is in contrast to the work by Nakijima et al.^32^ who reported that a lateral wedge insole with an arch support reduced medial knee loading more than a standard lateral wedge. One reason for this difference is that the lateral wedge insole with the medial support used in this study is an off‐the‐shelf device and not custom made as in the study by Nakajima et al. It is noteworthy that the medial support incorporated into our lateral insole was not hard and could readily be compressed with weight‐bearing and this may underlie the similar effects of both insoles we studied. Both insoles were deemed to be comfortable (Table 3) but the supported lateral wedge received a greater overall comfort score and significantly reduced pain immediately in comparison to the typical wedge.

The mechanism for these reductions in EKAM and KAAI are perceived to be related to the change in the centre of pressure location for the lateral wedge insoles^33^ which leads to a greater reduction in moment arm. The barefoot walking had a slightly slower speed but this was not associated with changes in EKAM or KAAI. Therefore, the mechanism for this is potentially due to altered foot mechanics but this was not the remit of this paper and further research is needed.

External knee flexion moments also contribute to medial knee loading and effects of shoe inserts or shoes on flexion moments could affect medial loading independently of EKAM or KAAI. We found no significant effects of shoe inserts or shoes on external knee flexion moment.^20^ Only barefoot walking reduced flexion moments and this may have been a consequence of a slower overall walking speed but this needs to be further explored. Further, recent work by Trepczynski and colleagues^8^ using instrumented knee prostheses suggests that the external knee flexion moment contributes importantly to medial knee loading only during activities when the knee is overly‐flexed, such as stair climbing and squatting or kneeling. Our participants were only required to walk on level ground and our findings on flexion moments suggest that with this activity, most of the variance in medial loading is readily explained by the EKAM and KAAI.

Our results on the effects of the mobility shoe contrast with earlier studies in that we found a reduction of just greater than 1% in medial knee loading during early stance. One possible reason could have been the choice of control shoe. As noted earlier Shakoor and cowokers^16^ tested mobility shoes against the individuals' own shoes. Those authors comment that the choice of shoe worn by the patient has an effect on reduction in medial knee loads compared with the mobility shoe. It is also possible that medial loading reductions occur over time with the mobility shoe.^34^ While the mobility shoe did not show expected reductions in medial loading, the participant's immediate knee pain scores were significantly improved in comparison to the control shoe with a favourable comfort rating. This suggests that patient adherence would be high and if medial loading were reduced significantly over time, this could be an effective intervention.

The reductions in pain seen in the mobility shoe and the lateral wedge insoles disagree with some longitudinal studies and the full reason behind why there were these changes in not known. However, one of the potential reasons could be due to an increased comfort in both the supported insole and the mobility shoe which reflected better perceived pain scores.

As with any study there are limitations other than the ones described earlier. The pain and comfort responses were assessed immediately and it is possible that these may change over time. However, previous work^35^ has suggested that immediate pain response and longer term pain response with wedges are highly correlated.

In conclusion, different lateral wedge insoles show comparable reductions in medial knee loading with the supported insole reducing pain more. In our study, the mobility shoe did not affect medial loading. While we confirmed findings of other studies in demonstrating a clearcut reduction in early stance medial loading when walking barefoot, barefoot walking increased medial loading during the latter period of stance and may not be the best for medial loading reduction.

## AUTHORS' CONTRIBUTIONS

All authors contributed to the study design, collection, analysis and editing and approval of the final manuscript.

## Supporting information

Additional supporting information may be found in the online version of this article.

**Supporting Information Table S1**: EKAM 1st Peak Post‐Hoc Pairwise Comparisons.
**Supporting Information Table S2**: EKAM 2nd Peak Post‐Hoc Pairwise Comparisons.
**Supporting Information Table S3**: KAAI Post‐Hoc Pairwise Comparisons.
**Supporting Information Table S4**: Max Flexor Moment Post‐Hoc Pairwise Comparisons.

## References

[1] Guccione AA , Felson DT , Anderson JJ , et al. 1994 The effects of specific medical conditions on the functional limitations of elders in the Framingham Study. Am J Public Health 84:351–358. 8129049 10.2105/ajph.84.3.351 PMC1614827

[2] Osteoarthritis. Care and management of osteoarthritis in adults. NICE 2014; Clinical Guideline 177.

[3] S. Ahlback , Osteoarthrosis of the knee. A radiographic investigation Acta Radiol Diagn (Stockh) 1968; 72 5706059

[4] Amin S , Luepongsak N , Mcgibbon CA , et al. Knee adduction moment and development of chronic knee pain in elders. Arthritis Rheum 2004; 51; 371–6. 15188321 10.1002/art.20396

[5] Miyazaki T , Wada M , Kawahara H , et al. Dynamic load at baseline can predict radiographic disease progression in medial compartment knee osteoarthritis. Ann Rheum Dis 2002; 61, 617–622. 12079903 10.1136/ard.61.7.617 PMC1754164

[6] OD Schipplein , TP. Andriacchi , Interaction between active and passive knee stabilizers during level walking J Orthop Res 9 1991; 113–119 1984041 10.1002/jor.1100090114

[7] Zhao D , Banks SA , Mitchell KH , et al. Correlation between the knee adduction torque and medial contact force for a variety of gait patterns. J Orthop Res 2007; 25, 789–97. 17343285 10.1002/jor.20379

[8] Trepczynski A , Kutzner I , Bergmann G , et al. Modulation of the relationship between external knee adduction moments and medial joint contact forces across subjects and activities. Arthritis Rheumatol 2014; 66, 5, 1218–1227 24470261 10.1002/art.38374 PMC4158863

[9] Mundermann A , Dyrby CO , Andriacchi TP. Secondary gait changes in patients with medial compartment knee osteoarthritis: increased load at the ankle, knee and hip during walking. Arthritis Rheum 2005; 52:2835–44 16145666 10.1002/art.21262

[10] Stefanyshyn DJ , Stergiou P , Lun VM et al. Knee angular impulse as a predictor of patellofemoral pain in runners. Am J Sports Med 2006; 34:1844–1851. 16735584 10.1177/0363546506288753

[11] Kito N , Skinkoda K , Yamasaki T , et al. Contribution of knee adduction moment impulse to pain and disability in Japanese women with medial knee osteoarthritis. Clin Biomech 2010; 25:914–919. 10.1016/j.clinbiomech.2010.06.008 20650554

[12] Creaby MW , Wang Y , Bennell KL et al. Dynamic knee loading is related to cartilage defects and tibial plateau bone area in medial knee osteoarthritis. Osteoarthritis Cartilage 2010; 18:1380–5. 20816980 10.1016/j.joca.2010.08.013

[13] Bennell KL , Bowles KA , Wang Y et al. Higher dynamic medial knee load predicts greater cartilage loss over 12 months in medial knee osteoarthritis. Ann Rheum Dis. 2011; 70:1770–4. 21742637 10.1136/ard.2010.147082

[14] RK Jones , CJ Nester , JD Richards , et al. A comparison of the biomechanical effects of valgus knee braces and lateral wedged insoles in patients with knee osteoarthritis Gait Posture. 37 2013; 368–372 22920242 10.1016/j.gaitpost.2012.08.002

[15] Pollo FE , Otis JC , Backus SI et al. Reduction of medial compartment loads with valgus bracing of the osteoarthritic knee. Am J Sports Med 2002; 30:414–21. 12016084 10.1177/03635465020300031801

[16] Shakoor N , Lidtke RH , Sengupta M et al. Effects of specialized footwear on joint loads in osteoarthritis of the knee. Arthritis Rheum. 2008; 59:1214–20. 18759313 10.1002/art.24017 PMC3653288

[17] CO Kean , KL Bennell , TV Wrigley , et al. Modified walking shoes for knee osteoarthritis: Mechanisms for reductions in the knee adduction moment J Biomech. 46 2013; 2060–2066 23768609 10.1016/j.jbiomech.2013.05.011

[18] Jones RK , Chapman GJ , Forsythe L et al. The Relationship between reductions in knee loading and immediate pain response whilst wearing lateral wedged insoles in knee osteoarthritis. J Orthop Res. 2014; 32:1147–1154. 24903067 10.1002/jor.22666 PMC4372252

[19] Erhart‐Hledik JC , Elspas B , Giori NJ , et al. Effect of variable‐stiffness walking shoes on knee adduction moment, pain, and function in subjects with medial compartment knee osteoarthritis after 1 year. J Orthop Res. 2012; 30:514–21. 21953877 10.1002/jor.21563

[20] Walter JP , D'Lima DD , Colwell CW Jr , et al. Decreased knee adduction moment does not guarantee decreased medial contact force during gait. J Orthop Res. 2010; 28:1348–54. 20839320 10.1002/jor.21142 PMC2984615

[21] Hinman RS , Bowles KA , Payne C , et al. Effect of length on laterlly‐wedged insoles in knee osteoarthritis. Arthritis Rheum. 2008; 59:144–147 18163399 10.1002/art.23249

[22] Jones RK , Zhang M , Laxton P , et al. The biomechanical effects of a new design of lateral wedge insoles on the knee and ankle during walking. Hum Mov Sci 32 2013; 596–604 24054897 10.1016/j.humov.2012.12.012

[23] Roos EM , Roos PH , Lohmander LS et al. Knee injury and Osteoarthritis Outcome Score (KOOS): development of a self‐administered outcome measure. J Orthop Sports Phys Ther 1998; 78:88–96. 9699158 10.2519/jospt.1998.28.2.88

[24] Altman RD , Gold GE. Atlas of individual radiographic features in osteoarthritis, revised. Osteoarthritis Cartilage 2007; 15 Supple A: A1–56. 17320422 10.1016/j.joca.2006.11.009

[25] Kerrigan DC , Lelas JL , Goggins J , et al. Effectiveness of a lateral‐wedge insole on knee varus torque in patients with knee osteoarthritis. Arch Phys Med Rehabil. 2002; 83, 889–893. 12098144 10.1053/apmr.2002.33225

[26] Cappozzo A , Catani F , Croce UD , et al. Position and orientation in space of bones during movement: anatomical frame definition and determination. Clin Biomech. 1995; 10 **,** 171–178. 10.1016/0268-0033(95)91394-t 11415549

[27] Bell AL , Brand RA , Pedersen DR . Prediction of hip joint centre location from external landmarks. Hum Mov Sci. 1989; 8:3–16.

[28] Glickman ME , Rao SR , Schulz MR. False discovery rate control is a recommended alternative to Bonferroni‐type adjustments in health studies. J Clin Epidemiol, 2014; 67:850–857 24831050 10.1016/j.jclinepi.2014.03.012

[29] Benjamini Y , Yekutieli D. False discovery rate controlling confidence intervals for selected parameters. J Am Stat Assoc, 2012; 100:71–80

[30] MJ Parkes , N Maricar , M Lunt . et al. Lateral wedge insoles as a conservative treatment for pain in patients with medial knee osteoarthritis: a meta‐analysis. JAMA 10 2013; 722–730 23989797 10.1001/jama.2013.243229 PMC4458141

[31] Shakoor N , Block JA. Walking barefoot decreases loading on the lower extremity joints in knee osteoarthritis. Arthritis Rheum. 2006 Sep;54: 2923–7. 16947448 10.1002/art.22123

[32] Nakajima K , Kakihana W , Nakagawa T et al. Addition of an arch support improves the biomechanical effect of a laterally wedged insole. Gait Posture, 2009; 29, 208–213. 18824355 10.1016/j.gaitpost.2008.08.007

[33] Hinman RS , Bowles KA , Meclaf BB et al. Lateral wedge insoles for medial knee osteoarthritis: Effects of lower limb frontal plane biomechanics. Clin Biomech, 2012; 27:27–33. 10.1016/j.clinbiomech.2011.07.010 21862189

[34] Shakoor N , Lidtke RH , Wimmer MA et al. Improvement in knee loading after use of specialized footwear for knee osteoarthritis: results of a six‐month pilot investigation. Arthritis Rheum 2013 May;65: 1282–9. 23575871 10.1002/art.37896

[35] Hinman RS , Payne C , Metcalf BR , et al. Lateral wedges in knee osteoarthritis: what are their immediate clinical and biomechanical effects and can these predict a three‐month clinical outcome. Arthritis Care Res Mar 59 408–415 10.1002/art.23326 18311763

