# Supplementary material for: The effect of different types of insoles or shoe modifications on medial loading of the knee in persons with medial knee osteoarthritis: a randomised trial
Source: J Orthop Res. 2015 Jun 3;33(11):1646–54. doi: 10.1002/jor.22947 (PMC4737200; doi:10.1002/jor.22947)
Supplement: Supplementary file 1 — Supporting Information Table S1: EKAM 1st Peak Post‐Hoc Pairwise Comparisons. Supporting Information Table S2: EKAM 2nd Peak Post‐Hoc Pairwise Comparisons. Supporting Information Table S3: KAAI Post‐Hoc Pairwise Comparisons. Supporting Information Table S4: Max Flexor Moment Post‐Hoc Pairwise Comparisons. [file JOR-33-1646-s001.docx]

**Supplementary Material**

**eTable 1: EKAM 1^st^ Peak Post-Hoc Pairwise Comparisons**

| **Contrast** | **Mean Difference** | **Unadjusted 95% CI** | **Unadjusted p** | **Benjamini-Hochberg Adjusted 95% CI** | **Benjamini-Hochberg Adjusted p (q-value)** |
| --- | --- | --- | --- | --- | --- |
| Typical vs Control | -0.023 | (-0.035 to -0.012) | <0.001 | (-0.038 to -0.008) | <0.001 |
| Supported vs Control | -0.022 | (-0.035 to -0.009) | 0.001 | (-0.039 to -0.006) | 0.003 |
| Mobility vs Control | -0.006 | (-0.021 to 0.008) | 0.384 | (-0.021 to 0.008) | 0.427 |
| Barefoot vs Control | -0.030 | (-0.044 to -0.016) | <0.001 | (-0.051 to -0.009) | <0.001 |
| Supported vs Typical | 0.001 | (-0.011 to 0.013) | 0.889 | (-0.011 to 0.013) | 0.889 |
| Mobility vs Typical | 0.017 | (0.001 to 0.032) | 0.032 | (-0.001 to 0.034) | 0.063 |
| Barefoot vs Typical | -0.007 | (-0.022 to 0.008) | 0.372 | (-0.023 to 0.009) | 0.427 |
| Mobility vs Supported | 0.016 | (0.001 to 0.031) | 0.042 | (-0.001 to 0.033) | 0.071 |
| Barefoot vs Supported | -0.008 | (-0.024 to 0.009) | 0.347 | (-0.025 to 0.010) | 0.427 |
| Barefoot vs Mobility | -0.024 | (-0.038 to -0.009) | 0.002 | (-0.041 to -0.006) | 0.004 |

**eTable 2: EKAM 2^nd^ Peak Post-Hoc Pairwise Comparisons**

| **Contrast** | **Mean Difference** | **Unadjusted 95% CI** | **Unadjusted p** | **Benjamini-Hochberg Adjusted 95% CI** | **Benjamini-Hochberg Adjusted p** |
| --- | --- | --- | --- | --- | --- |
| Typical vs Control | -0.028 | (-0.036 to -0.020) | <0.001 | (-0.039 to -0.016) | <0.001 |
| Supported vs Control | -0.018 | (-0.026 to -0.010) | <0.001 | (-0.028 to -0.008) | <0.001 |
| Mobility vs Control | -0.005 | (-0.015 to 0.005) | 0.294 | (-0.015 to 0.005) | 0.326 |
| Barefoot vs Control | 0.001 | (-0.011 to 0.013) | 0.856 | (-0.011 to 0.013) | 0.856 |
| Supported vs Typical | 0.010 | (0.002 to 0.017) | 0.013 | (0.001 to 0.018) | 0.022 |
| Mobility vs Typical | 0.023 | (0.012 to 0.033) | <0.001 | (0.010 to 0.035) | <0.001 |
| Barefoot vs Typical | 0.029 | (0.018 to 0.040) | <0.001 | (0.014 to 0.044) | <0.001 |
| Mobility vs Supported | 0.013 | (0.002 to 0.023) | 0.017 | (0.002 to 0.024) | 0.024 |
| Barefoot vs Supported | 0.019 | (0.007 to 0.031) | 0.002 | (0.005 to 0.033) | 0.004 |
| Barefoot vs Mobility | 0.006 | (-0.004 to 0.017) | 0.243 | (-0.005 to 0.017) | 0.304 |

**eTable 3: KAAI Post-Hoc Pairwise Comparisons**

| **Contrast** | **Mean Difference** | **Unadjusted 95% CI** | **Unadjusted p** | **Benjamini-Hochberg Adjusted 95% CI** | **Benjamini-Hochberg Adjusted p (q-value)** |
| --- | --- | --- | --- | --- | --- |
| Typical vs Control | -0.012 | (-0.016 to -0.009) | <0.001 | (-0.017 to -0.008) | <0.001 |
| Supported vs Control | -0.009 | (-0.013 to -0.005) | <0.001 | (-0.014 to -0.003) | <0.001 |
| Mobility vs Control | -0.004 | (-0.009 to 0.001) | 0.090 | (-0.010 to 0.001) | 0.129 |
| Barefoot vs Control | -0.007 | (-0.013 to -0.001) | 0.023 | (-0.014 to 0.000) | 0.057 |
| Supported vs Typical | 0.004 | (0.000 to 0.008) | 0.064 | (-0.001 to 0.008) | 0.107 |
| Mobility vs Typical | 0.008 | (0.003 to 0.014) | 0.003 | (0.001 to 0.015) | 0.011 |
| Barefoot vs Typical | 0.006 | (0.000 to 0.011) | 0.042 | (-0.001 to 0.012) | 0.085 |
| Mobility vs Supported | 0.004 | (-0.001 to 0.010) | 0.110 | (-0.001 to 0.01) | 0.138 |
| Barefoot vs Supported | 0.002 | (-0.004 to 0.008) | 0.509 | (-0.004 to 0.008) | 0.509 |
| Barefoot vs Mobility | -0.002 | (-0.008 to 0.004) | 0.428 | (-0.009 to 0.004) | 0.476 |

**eTable 4: Max Flexor Moment Post-Hoc Pairwise Comparisons**

| **Contrast** | **Mean Difference** | **Unadjusted 95% CI** | **Unadjusted p** | **Benjamini-Hochberg Adjusted 95% CI** | **Benjamini-Hochberg Adjusted p (q-value)** |
| --- | --- | --- | --- | --- | --- |
| Typical vs Control | -0.002 | (-0.022 to 0.018) | 0.818 | (-0.022 to 0.018) | 0.818 |
| Supported vs Control | 0.013 | (-0.004 to 0.030) | 0.133 | (-0.005 to 0.032) | 0.190 |
| Mobility vs Control | -0.006 | (-0.029 to 0.017) | 0.611 | (-0.030 to 0.018) | 0.764 |
| Barefoot vs Control | -0.035 | (-0.057 to -0.013) | 0.002 | (-0.064 to -0.006) | 0.009 |
| Supported vs Typical | 0.016 | (-0.002 to 0.033) | 0.078 | (-0.004 to 0.035) | 0.156 |
| Mobility vs Typical | -0.004 | (-0.027 to 0.019) | 0.754 | (-0.027 to 0.020) | 0.818 |
| Barefoot vs Typical | -0.033 | (-0.054 to -0.011) | 0.003 | (-0.059 to -0.006) | 0.009 |
| Mobility vs Supported | -0.019 | (-0.043 to 0.005) | 0.115 | (-0.046 to 0.007) | 0.190 |
| Barefoot vs Supported | -0.048 | (-0.07 to -0.026) | <0.001 | (-0.079 to -0.017) | <0.001 |
| Barefoot vs Mobility | -0.029 | (-0.05 to -0.008) | 0.008 | (-0.054 to -0.004) | 0.019 |
